# Decoding odor responses: universal patterns and individual signatures in psychophysiology using nonlinear models

**DOI:** 10.1093/chemse/bjag009

**Published:** 2026-03-05

**Authors:** Tim L Jesgarzewsky, Antonie L Bierling, Ilona Croy

**Affiliations:** Department of Clinical Psychology, Friedrich Schiller University of Jena, Jena, Germany; Department of Clinical Psychology, Friedrich Schiller University of Jena, Jena, Germany; Clinic and Polyclinic for Psychotherapy and Psychosomatics, Faculty of Medicine and University Hospital Carl Gustav Carus, TUD Dresden University of Technology, Dresden, Germany; Institute of Materials Science, Technische Universität Dresden, Dresden, Germany; Department of Clinical Psychology, Friedrich Schiller University of Jena, Jena, Germany; German Center for Mental Health (DZPG), Site Jena-Magdeburg-Halle, Jena, Germany

**Keywords:** intensity, nonlinear modeling, olfaction, perception, psychophysiology, valence

## Abstract

Olfactory perception is a complex process driven by the chemical properties of odorants and shaped by a multitude of individual factors. As a result, predicting how an individual perceives a given odor remains challenging. We aimed to address this complexity by integrating individual response patterns, odorant properties, and psychophysiological responses into a unified model. Therefore, we tested perceptual dimensions (valence, temperature, and intensity) of 6 perceptually diverse monomolecular odorants with continuous time-series data from psychophysiological measures (respiration, heart rate, electromyography [EMG] corrugator, and EMG zygomaticus) in a sample of 41 participants. By simultaneously accounting for the odorant itself, individual rating tendencies, and both group-level and individual-specific physiological effect patterns in the nonlinear modeling process, we found that while the specific odorant and individual rating tendencies were the primary drivers of perception, the relative contributions varied significantly across perceptual dimensions. The inclusion of physiological signals significantly improved the predictive models, revealing that both generalizable (group-level) and highly individualized psychophysiological response patterns contributed to how an odor was perceived. Examination of the specific effect patterns revealed respiration and EMG corrugator as key group-level predictors for valence and intensity, while significant individual-specific effect patterns varied considerably across the perceptual dimensions. Our findings demonstrate that a comprehensive understanding of olfactory perception requires the consideration of the interplay between stimulus characteristics, idiosyncratic biases, and distinct universal versus person-specific physiological signatures, offering a more nuanced understanding of this sensory experience.

## 1. Introduction

The perception of an odor is a multifaceted sensory experience that elicits a range of responses, from triggering memories and emotions to influencing behavior (Willander and Larsson 2007; Stevenson 2010). Understanding the intricate relationship between the chemical structure of an odorant and how the individual perceives it, often referred to as the “stimulus-percept problem,” is a fundamental challenge in olfactory research (for a review, see Auffarth 2013).

This complexity arises from several factors, including the nature of the olfactory system itself. At the receptor level, the human olfactory system comprises a total repertoire of around 400 functional olfactory receptor genes (Olender et al. 2008; Niimura et al. 2014). Each receptor can bind to a variety of odorant molecules, and conversely, each odorant can activate multiple receptor types. This intricate interplay results in a combinatorial code, where the pattern of activated receptors, rather than any individual receptor, determines the perceived odor identity (Buck 2004; Auffarth 2013; del Mármol et al. 2021).

Adding to this complexity is the sheer number of potential odorants, estimated to exceed 40 billion unique compounds (Mayhew et al. 2022), each with a unique combination of molecular properties capable of activating a specific set of olfactory receptors. Even subtle variations in these molecular properties, such as a different chirality, can profoundly alter an odor's perceptual qualities. Some functional groups are associated with specific notions, such as esters typically evoking a fruity or floral impression. This association, however, only holds for a small set of groups, meaning that it is generally difficult to infer the percept of an odorant based on its molecular structure (Auffarth 2013; Secundo et al. 2014; Genva et al. 2019).

Anyhow, odor perception can be predicted with some success. For instance, Keller et al. (2017) showed that chemical properties relate to how people describe the pleasantness, intensity, and some other odor-evoked olfactory impressions. This interesting result, obtained with machine learning approaches, did however only hold for the group level. The algorithm failed to consistently predict how an individual describes an odor.

Due to the large influence of interindividual differences, it is a typical finding that the stimulus-percept relation is more accurate and stable when analyzed at the group level (Dravnieks 1982; Bierling et al. 2021). Group-level averages are therefore considered a way to establish a ground-truth of perceptual properties of odorants (Lee et al. 2023) and studies often strive to minimize individual differences that shape olfactory experiences. Besides group-averaging, another approach to reduce interindividual variability is to utilize expert panels, such as perfumers, or trained raters to evaluate perceptual properties (Zarzo 2015; Lee et al. 2023). These methods are particularly useful for assessing group-level relationships between molecular and perceptual properties as it enhances rater reliability (Dravnieks 1982), but it discards potentially relevant interindividual variability.

Indeed, approximately 66% of potentially functional olfactory receptors are expressed in each person, though individual variation is substantial (Zhang et al. 2007; Verbeurgt et al. 2014). This interindividual variance in receptor expression is in line with interindividual variations in detectable odors (Croy et al. 2015) and overall perceptual responses (Trimmer et al. 2019).

How we perceive an odor is further influenced by a multitude of factors, including age, sex, genetics, cultural background, and prior experiences (Ayabe-Kanamura et al. 1998; Chrea et al. 2004; Keller et al. 2012; Croy et al. 2015; Fjaeldstad et al. 2017; Trimmer et al. 2019). Consequently, sometimes vast individual differences in odor perception result in diametral positioned ratings of pleasantness and intensity for the same odors (Bierling et al. 2021) and that some individuals even rate generally considered unpleasant odors, like feces, as more pleasant than generally considered pleasant odors, like vanilla (Croy et al. 2014).

In order to understand the relation between odor and percept, we need to detail the olfactory percept itself. A comprehensive set of queried properties typically includes pleasantness, intensity, disgust, edibility, familiarity, irritability, and perceived temperature (Keller and Vosshall 2016; Bierling et al. 2025), with pleasantness and edibility often reported to be predominant characteristic of odorants explaining the most variance (Auffarth 2013; Secundo et al. 2014). Many of these perceptual ratings are related to a basal appraisal of approach or avoidance toward the source of the odor. These fundamental motivational schemes suit the functions of olfaction, such as evaluating food sources, social communication, and hazard avoidance (Stevenson 2010) and are reflected in autonomous responses to odors, such as heart rate and respiration, and perceptual odor properties.

For heart rate, odor pleasantness relates to a decreased heart rate variability while unpleasant odors relate to an increased heart rate (Bensafi et al. 2002a; He et al. 2014). In terms of respiration, a longer and deeper inhalation is positively related to perceived pleasantness of odors (Delplanque et al. 2009; Joussain et al. 2017). Facial electromyography (EMG), which measures muscle activity associated with emotional expressions, can provide further information. For example, an increased activity at the Corrugator Supercilii for unpleasant/negative valent and disgusting odors (Bensafi et al. 2002b; Delplanque et al. 2009; Pichon et al. 2015) and an increased activity at the Zygomaticus Major for pleasant odors (Pichon et al. 2015). However, not all studies showed a coherent relation of all physiological channels and perceptual properties (compare Delplanque et al. 2009; Pichon et al. 2015; Joussain et al. 2017, regarding zygomaticus, heart rate, and respiration, respectively).

Psychophysical patterns can consistently differ between individuals. As for rating data, where some persons tend to be more conservative or liberal in their evaluation of an odor, some persons tend to be more or less expressive in their mimical display, more or less variable in their heart rate, and exhibit different respiration patterns. Hence, individual patterns may improve the prediction of odor perception.

However, this requires advanced statistical approaches and has—to the best of our knowledge—not yet been done. An initial step toward more nuanced modeling is the inclusion of temporal dynamics. The initial heart rate increase peaking around 3 s after respiration onset, is, for instance, linked to novelty detection and arousal, while the following heart rate deceleration with its minimum around 6 s after respiration onset, is associated with valence assessments and odor differentiation (Delplanque et al. 2009; He et al. 2014; Pichon et al. 2015).

Capturing such nuances is challenging as it requires many repetitions of odor presentation to allow *α*-level correction in multiple testing and as the linear assumptions underlying traditional statistical models are not suited to analyze nonlinear psychophysiological signals. Maybe therefore, psychophysiological approaches in olfactory research seemingly led to an impasse in the last 10 years. Advances in analytical approaches make it worthwhile to investigate the dynamic interplay between psychophysiological measurements and odor perception in more detail under consideration of temporal dynamics and at the level of the individual.

We aimed to better understand the interplay of physiological responses and perception evoked by specific odorants. To do this, we used predictive nonlinear modeling techniques to examine the individual accuracy of odor perception by modeling odor, time-dependent physiology on the group level, individual rating bias, and individual physiology.

## 2. Materials and methods

### 2.1 Participants

A total of 66 German-speaking participants were recruited via an email newsletter and advertisement in lectures for students in BSc Psychology. Exclusion criteria were chronic or acute rhino-nasal impairments. Inclusion criteria were subjective health and normosmic function indicated by a score greater than 11 on the Sniffin’ Sticks Identification Test (Kobal et al. 1996; Hummel et al. 1997). Nine participants were excluded based on the identification test, resulting in a sample size of 57 participants (mean age = 21.9, SD = 2.4, range: [18, 27]; sex: 49 females, 8 males). Most participants (*n* = 50) reported at least 1 COVID infection in the past and none reported any ongoing infection. The study was conducted in accordance with the Declaration of Helsinki and was approved by the ethics board of the Friedrich Schiller University Jena (FSV 23/019).

### 2.2 Procedure

Participants were instructed to refrain from eating or smoking half an hour before arrival in the lab. After obtaining informed consent and assessing olfactory function, participants evaluated the quantitative and qualitative perceptual properties of 6 monomolecular odorants. At the same time, psychophysiological measurements of respiration, ECG, and facial EMG at the muscle sites Corrugator Supercilii and Zygomaticus Major were recorded using the recording device NIRx Wings with a sampling frequency of 500 and 131 Hz low-pass hardware filter. The placement of the electrode for respiration, ECG, and the reference electrode were done following the manufacturer's instructions. The EMG electrodes were placed following the guidelines in Fridlund and Cacioppo (1986). In addition, participants' verbal skills were tested prior to the experiment, which is beyond the scope of the present study.

#### 2.2.1 Odorants

**Figure 1:**
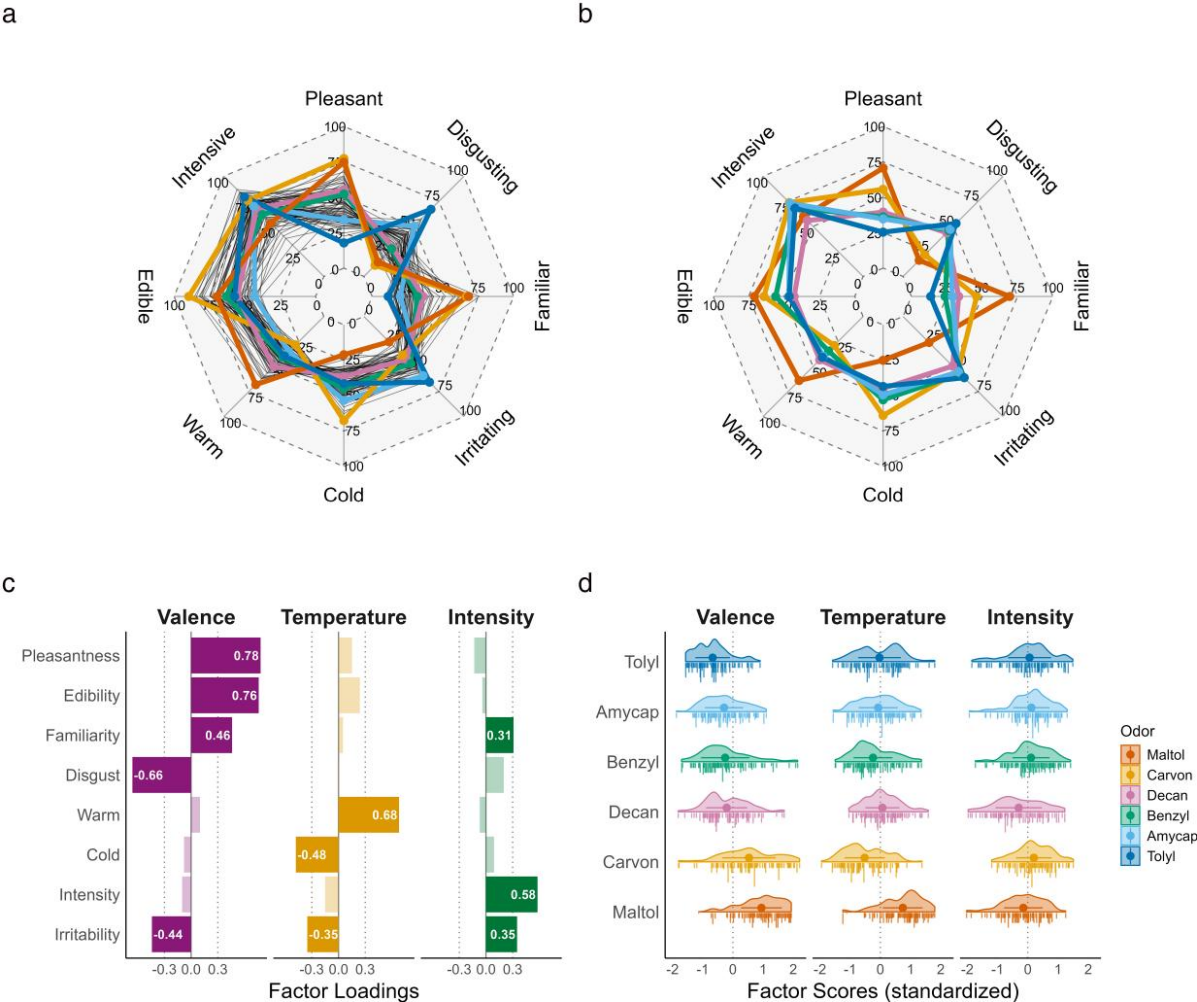
Selected odors and rating patterns. a) Average perceptual ratings of the 74 odorants in the original study (Bierling et al. 2025). Black lines visualize the ratings of all 74 odors, colorized lines depict the 6 selected odors. The odors were chosen based on their wide and diverse coverage of the rating value spectrum on all considered perceptual properties. b) Average perceptual ratings of the selected odorants in the current study confirmed the initial selection. c) Rating properties were reduced by EFA resulting in 3 perceptual dimensions, the loadings > 0.3 of the varimax-rotated factors are displayed and d) the distribution of the resulting factor scores shows a distinct perceptual pattern across the selected odors. Ratings for a) and b) were given on a VAS, ranging from 0 (“Does not fit at all”) to 100 (“Fits very well”). Point ranges in d) depict means and standard deviations.

Out of the 74 odors presented in Bierling et al. (2025), a subset of 6 odorants was selected (Table 1) which cover a wider response spectrum on most of the perceptual properties, namely pleasantness, disgust, edibility, irritation, cold, warm, familiarity, and intensity (Fig. 1a). To ensure that odor perception is not superimposed by intensity, we aimed to present each odor at approximately isointense concentrations perceived around 75% intense (informed by the concentrations used in Bierling et al. 2025). Dipropylene glycol (CAS-Number: 25265-71-8) was used as a solvent for all odorants. As initial testing of the study procedure indicated that this concentration is too weak to evoke a reliable percept when presented with the olfactometer, in comparison to odor presentation via direct sniffing and unrestricted sniffing duration as done in Bierling et al. (2025), we increased the concentration by 1 order of magnitude for all odors. Table 1 depicts the concentrations after adjusting for the presentation with the olfactometer.

**Table 1:** Odorants presented in this study.

Short names	Trivial name	CAS-No.	Concentration
**Amycap**	Amyl caproate	540-07-8	Undiluted
**Maltol**	Maltol	118-71-8	1/10
**Benzyl**	Benzyl acetate	140-11-4	1/10
**Decan**	4-Decanolide	706-14-9	1/1
**Carvone**	*R*-(−)-Carvone	6485-40-1	1/1
**Tolyl**	*p*-Tolyl acetate	140-39-6	1/10

Odorants were diluted in Dipropylene Glycol (CAS-No. 25265-71-8). Concentrations are expressed as volume/volume ratios (odorant:solvent).

#### 2.2.2 Experimental procedure

The odorants were presented binasaly using the olfactometer Sniff-0 (Cynexo srl) with an airflow of 2 standard liters per minute (SLPM) via an air headspace delivery system A constant, positive pressure airflow of 2 SLPM was maintained for all channels. Each odorant channel was routed through separate polytetrafluoroethylene (PTFE) tubes (6 mm diameter, ∼3 m length). These tubes converged in a manifold, from which 2 final PTFE tubes (4 mm diameter) were inserted into each nostril of the participant. Fast-acting ON–OFF solenoid valves (4 ms activation time) controlled the clean air flow for each channel, ensuring precise, pulsed odor presentations.

The experiment was presented using Psychopy (Peirce et al. 2019). All instructions and perceptual properties were originally presented in German, but are reported here in English for convenience. Instructions were given via a monitor. Each trial began with a 5-s visual countdown, after which participants were instructed to breathe with odor onset (“Inhale in [countdown]…”), followed by the 3,000 ms odor presentation. Immediately following the 3,000 ms presentation, participants rated one of the 8 perceptual properties on a visual analog scale (VAS) ranging from 0 (“Does not fit at all”) to 100 (“Fits very well”). Ratings were performed using a mouse cursor to drag an indicator on the scale and confirm the selection with a button press. The interstimulus interval (ISI) was self-paced, lasting a minimum of 15 s, and only terminated once the participant completed the rating. A constant flow of clean air was presented during the ISI. Prior to the experiment, the procedure was explained, questions were answered, and a practice trial with 1 random odorant and perceptual property was conducted. Due to hardware limitations, the 6 odorants were split into 2 blocks of 3, with the odorants in each block remaining consistent across participants, while the block order was randomized. Within each block, each odorant was presented with each of the 8 perceptual properties in a fully randomized order. The 8 properties rated were (original German question in parentheses): Pleasantness (“Wie angenehm war der Geruch?”), Disgust (“Wie Ekel erregend war der Geruch?”), Edibility (“Wie essbar war der Geruch?”), Irritability (“Wie reizend war der Geruch?”), Familiarity (“Wie mir bekannt war der Geruch?”), Intensity (“Wie intensiv war der Geruch?”), Warmth (“Wie warm war der Geruch?”), and Coldness (“Wie kalt war der Geruch?”). This design resulted in a total of 96 trials (6 odorants × 8 perceptual properties × 2 repetitions).

### 2.3 Statistical methods

#### 2.3.1 Examining latent structure of the perceptual ratings

Supporting our initial odor selection, the average ratings per odor were comparable to the previous study (Bierling et al. 2025, compare Fig. 1a and b; for a depiction of the distribution of perceptual ratings, see Supplementary Fig. A1 in the Supplementary Appendix). While the perceptual properties were selected to archive a differentiated picture of the percept and to keep comparability to Bierling et al. (2025), they entailed some redundancies: the ratings “warm” and “cold” or “pleasantness’ and “disgust,” for instance, depict different ends of a temperature and valence scale, respectively. To reduce the risk of *α*-error inflation, we condensed the information to its latent structure by computing an exploratory factor analysis (EFA) across all rating dimensions. Bartlett's test for sphericity ((28) = 1199,65, *P* < 0.001) and the Kaiser–Meyer–Olkin statistic (KMO = 0.78) both indicated that the data were suitable for a factor analysis. A total of 3 factor dimensions were selected using the R-package parameters (Lüdecke et al. 2020). Thus, a varimax-rotated EFA with 3 factors was computed (RMSEA < 0.01, TLI > 0.99) and trial-wise factor scores were extracted as variables of interest (see Fig. 1c and 1d).

Together, these 3 factors explained 44% of the variance, which is comparable to what other studies found for a similar number of factors or principal components (Khan et al. 2007; Dalton et al. 2008; Zarzo 2008; Zarzo and Stanton 2009). The first factor explained 24% and was mainly defined by the strong positive loading of pleasantness and a strong negative loading of disgust. It will, therefore, be referred to as “valence.” The second factor explained 11% and was “temperature-related,” with cold and (negatively) warm loading the highest onto this factor. The last factor, “intensity,” explained 9% with intensity and irritability as its highest loading perceptual properties.

#### 2.3.2 Psychophysiological signals

##### 2.3.2.1 Data preprocessing

The psychophysiological signals were preprocessed using the Python package ‘Neurokit2’ (Makowski et al. 2021). The raw ECG signal was filtered with a 0.5 Hz high-pass 5th-order Butterworth filter and a 50 Hz notch filter. The respiratory signal was linear detrended and filtered using a 5th-order 2 Hz low-pass IIR Butterworth filter. The fEMG signals were first baseline detrended, denoised using a 20 to 50 Hz 2nd-order band-pass Butterworth filter, followed by a 50 Hz notch filter. The signal was then rectified using a linear envelope. After the initial signal preprocessing, signals were split trial-wise with a duration of 15 s (thereof 1 s baseline prior to odor onset). Trial-wise visual inspection was performed to exclude trials with clear motion or electrode movement artifacts (1.1% of the data).

Respiration and heart rate data were each baseline-corrected. Heart rate was thereafter corrected for respiration-dependent effects. For this, a linear regression was computed with both, the concurrent and 1 frame lagged respiration, as independent variables and the concurrent heart rate as dependent variable. The residuals of this model were used as the corrected heart rate signal. To optimize computational efficiency, signals were downsampled from 500 to 5 Hz. Subsequently, participant-specific signal normalization was performed by dividing each psychophysiological signal separately by its respective standard deviation across time and trials. This was done to increase the comparability of variances between participants while still preserving the pattern of variability within each participant.

Drawing from the field of functional data analysis, we detected outliers by using the Fraiman and Muniz depth implemented in the R-package “fda.usc” (Febrero-Bande and de la Fuente 2012) to quantify the centrality of the time course in all trials for each psychophysiological signal separately. This method assigns a numerical depth score to each curve, allowing us to identify the least typical observations, defined by the lowest depth scores. We defined and subsequently removed outlier trials as the 1% of curves with the lowest depth scores. Participants with less than 8 trials for any of the 8 odorants after outlier exclusion were discarded from the analysis, resulting in a final sample size of *N* = 41 for the nonlinear analysis (mean age = 22.0, SD = 2.6, range: [18, 27]; 37 females, 4 males).

##### 2.3.2.2 Statistical analysis

In order to analyze time-dependent effects and to differentiate between group-level effects and individual effects, we employed a functional data analysis approach based on generalized additive models (GAMs) with scalar-on-function regressions and random effects. The GAM framework allows for the modeling of nonlinear relationships between psychophysiological measures and perceptual ratings. Scalar-on-function regression enables the inclusion of the entire time course of psychophysiological measures as independent predictors, respecting the continuous nature of the signal, preventing the necessity to adjust for multiple testing and avoiding the information loss associated with averaging (for more details regarding functional regression approaches, see Morris 2015). Given the importance of individual differences in odor perception, random effects were included to allow for trial-wise analysis and the separation of global and individual effects of the psychophysiological measures.

While alternative prediction methods, such as random-forest approaches, could be used to analyze the data, we decided against them. Our primary goal was not solely to predict olfactory perception, but to investigate the interplay of perceptual properties and the temporal dynamics of psychophysiological responses. Using GAMs allowed us to specifically model these temporal dynamics, making it easier to interpret the significance of each physiological signal and identify the exact time points where a signal is associated with a particular perceptual dimension.

For the computation of the scalar-on-function models, the implementation of generalized additive models with restricted maximum likelihood in the R-package “mgcv” was used (Wood 2011). The time-course data were split randomly across participants and trial into a training set, containing 75% of all trials, and a testing set, containing 25% of all trials. One model for each perceptual dimension resulting from the EFA was computed using the training set, with the perceptual dimension as the outcome. The included signal's duration was determined by comparing the goodness-of-fit measures Akaike information criterion (AIC) and Bayesian information criterion (BIC) and the explained deviance for models fitted exclusively on the training data with varying time spans for the psychophysiological signals, resulting in the selection of a signal duration of 6 s. The group-level effect of odor perception was incorporated as a categorical fixed effect, the group-level effect of each psychophysiological signal as linear functional on time using thin-plate regression splines, the individual rating tendency as random effect for each participant, which are modeled in “mgcv” as parametric terms penalized by a ridge penalty, and the individual effects of each psychophysiological signal as factor-smooth interaction of each participant with the linear functional on time. Additionally, the test half in which the trial occurred was included as fixed categorical covariate. The final evaluation of the models on the testing data indicated no remarkable overfit in terms of their root mean square error (RMSE) and adjusted determination coefficient (*R*^2^): valence: $R_{\mathrm{training}}^{2}$ = 0.557, $R_{\mathrm{testing}}^{2}$ = 0.508, RMSE_training_ = 0.590, RMSE_testing_ = 0.612; temperature: $R_{\mathrm{training}}^{2}$ = 0.567, $R_{\mathrm{testing}}^{2}$ = 0.483, RMSE_training_ = 0.482, RMSE_testing_ = 0.517; intensity: $R_{\mathrm{training}}^{2}$ = 0.499, $R_{\mathrm{testing}}^{2}$ = 0.447, RMSE_training_ = 0.496, RMSE_testing_ = 0.508. Further, all 3 models showed a better model fit in terms of AIC and BIC when compared with linear mixed models with time-averaged predictors (see Supplementary Table A1 in the Supplementary Appendix).

To determine the significance of individual physiological signals, group-level physiological signal effects, group-level odor perception, and individual rating tendencies, we employed sequential model comparisons. Specifically, we used analysis of deviance with an *F* statistic, as implemented in the “mgcv” R package, and examined the changes in *R*^2^ values between nested models. Predictors were systematically removed in the following sequence: all individual physiological signal effects, all group-level physiological signal effects, the individual rating tendency, and finally, the group-level odor perception. Furthermore, Wald-like tests, also conducted using the “mgcv” R package, were used to assess the statistical significance of individual physiological signal predictors within the final models. Visual inspection of partial effects along the time domain, with 95% pointwise confidence intervals, facilitated the identification of specific patterns of significant smooth effects. When a confidence interval did not include zero, it was considered noteworthy at that particular time point. Note, that the statistical significance of a smooth was assessed across the entire smooth, not on a pointwise level.

## 3. Results

We computed 3 nonlinear generalized additive models (GAMs) to separately predict the perceptual dimensions of odors (valence, temperature, intensity) based on the presented odor, individual rating tendencies, and the physiological response patterns (respiration, heart rate, EMG Corrugator, and EMG Zygomaticus). Physiological responses were modeled hierarchically on 2 levels: a group-level effect representing the general trend across participants, and an individual-level effect capturing the unique, consistent trend within each participant.

After consideration of all those factors, the perceptual dimensions of valence, temperature, and intensity were predicted with an accuracy of 50% to 56% (Table 2). Hence, despite exhaustive data collection and nuanced modeling, 50% to 44% of odor perception remained unexplained.

**Table 2:** Differences in adjusted $R^{2}$ of each model through successive removal of predictors.

	Valence (*R^2^* = 0.556)	Temperature (*R^2^* = 0.567)	Intensity (*R^2^* = 0. 499)
**w/o group-level odor**	−0.339***	−0.268***	−0.076***
**w/o individual rating tendency**	−0.155***	−0.237***	−0.354***
**w/o individual physiol. response**	−0.025***	−0.031***	−0.031***
**w/o group-level physiol. response**	−0.015***	−0.002	−0.014***

Total adjusted $R^{2}$ of each model are depicted in parentheses. Note that the differences in $R^{2}$ do not add up to the $R^{2}$ of the full models due to the involvement of intercepts and the remaining covariate. Asterisks indicate statistically significant *P* values in the sequential model comparison tests.

^***^ *P* < 0.001.

**Figure 2:**
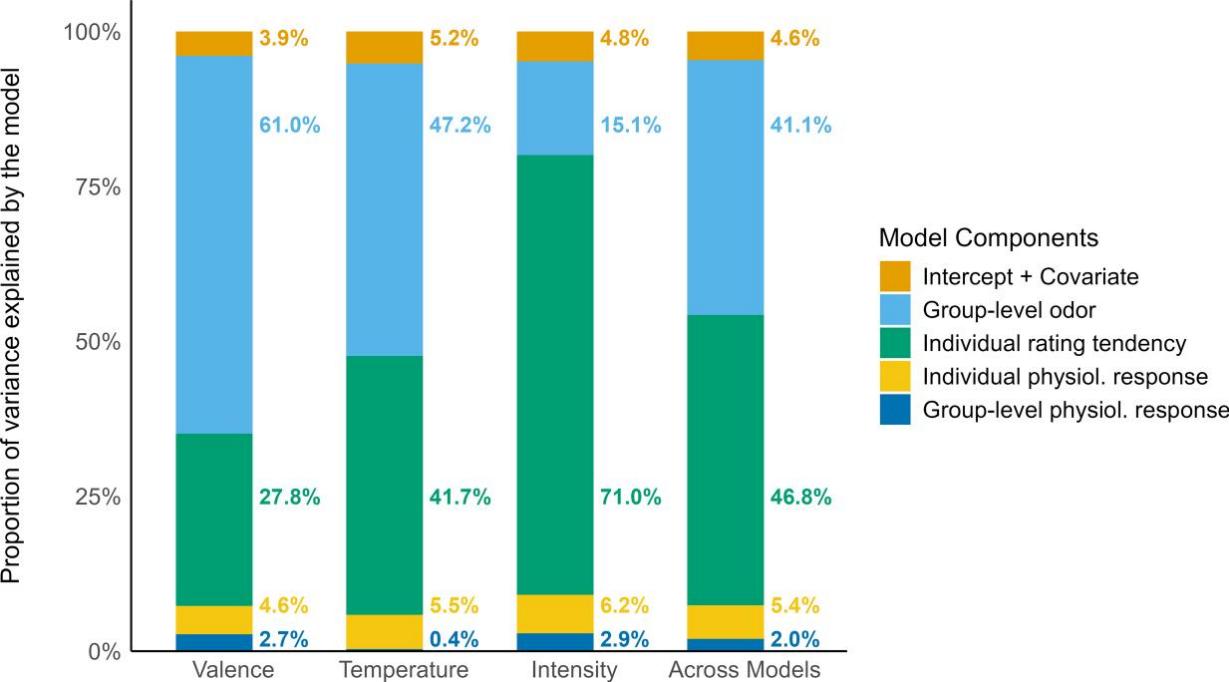
Proportions of explained variance (adjusted *R*^2^) attributable to specific model components within each of the separate models for the prediction of valence, temperature, and intensity. These proportions were calculated based on the difference in adjusted *R*^2^ resulting from the exclusion of the respective components. The final column displays the average proportion for the components, averaged across all models.

Of the variance explained by each model, the odor itself contributed 15.1% to 61%, with the highest proportion for valence and a lowest proportion for intensity (Fig. 2).

Individual rating patterns were about as important as the odor itself, ranging from 27.8% to 71% of the explained variance in the models while the time course of psychophysiological signals contributed a smaller portion of additional variance in the range of 0.4% to 2.9% on the group level and 4.6% to 6.2% on the individual level, depending on the perceptual dimension (see Table 2, Fig. 2).

### 3.1 Odor rating

There was a greater consistency in valence than intensity across participants for specific odors ($\Delta R^{2}$ = −0.339 vs. $\Delta R^{2}$ = −0.076), while within participants, intensity was more stable than valence ($\Delta R^{2}$ = −0.354 vs. $\Delta R^{2}$ = −0.155). Overall, the predictions of the presented odors showed a wide spread across the perceptual dimensions. Marginal estimates (Table 3; for participant-wise predictions, see Supplementary Table A2 in the Supplementary Appendix) indicated that Maltol was consistently rated as the most pleasant, warmest, and least intense odor. Conversely, Tolyl was judged as the most unpleasant, Carvon as the coldest, and Amycap as the most intense. Notably, while showing significant differences between the odorants, the intensity dimension exhibited the narrowest range between the lowest and highest marginal estimates (range = 0.5 when compared with 1.5 for valence and 1.2 for temperature), aligning in tendency with the intended iso-intensity of the odors.

**Table 3:** Predicted estimates of the odors on each perceptual dimension.

Odor	Valence	Temperature	Intensity
**Tolyl**	**−0.61** **[−0.67, −0.54]** **(−0.61)**	0.03 [−0.03, 0.09] (0.03)	0.12 [0.05, 0.18] (0.12)
**Amycap**	−0.25 [−0.32, −0.19] (−0.26)	−0.02 [−0.08, 0.03] (−0.02)	**0.22 [0.16, 0.28]** **(0.22)**
**Benzyl**	−0.21 [−0.28, −0.14] (−0.21)	−0.28 [−0.33, −0.22] (−0.28)	0.13 [0.07, 0.20] (0.13)
**Decan**	−0.20 [−0.27, −0.13] (−0.20)	0.17 [0.11, 0.23] (0.17)	−0.20 [−0.26, −0.13] (−0.20)
**Carvon**	0.56 [0.5, 0.63] (0.56)	**−0.48 [−0.54, −0.42]** **(−0.48)**	0.17 [0.11, 0.23] (0.17)
**Maltol**	**0.88 [0.81, 0.95]** **(0.88)**	**0.74 [0.67, 0.8]** **(0.73)**	**−0.28 [−0.35, −0.22]** **(−0.29)**

Values are marginal estimates with 95% confidence intervals in brackets and descriptive averages in parentheses. Bold values indicate the highest and lowest prediction on each perceptual dimension.

### 3.2 Psychophysiological responses to odors

**Figure 3:**
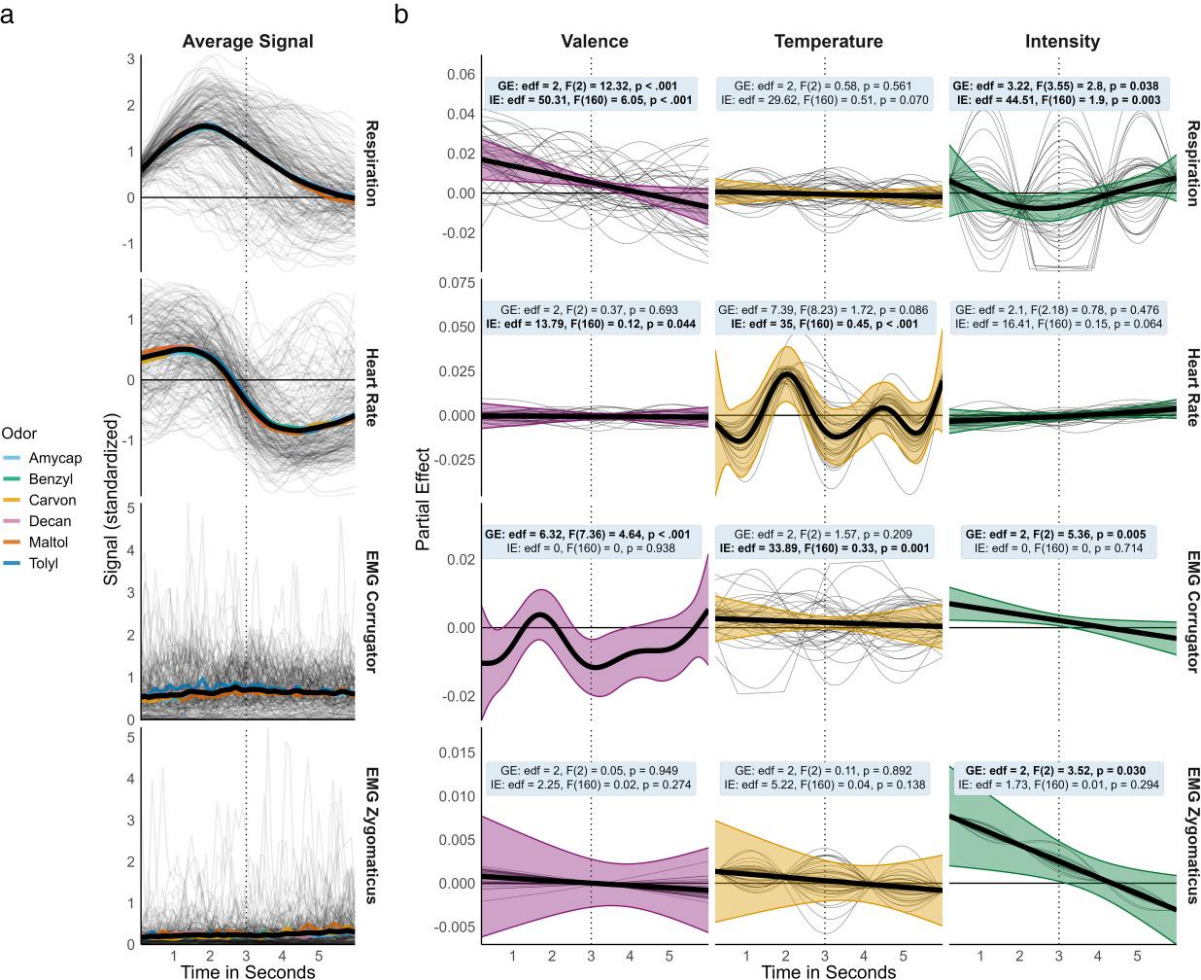
Psychophysiological signals and respective partial effect patterns during odor presentation (0 to 3 s; dotted vertical line: stimulus offset) and a 3 s post-stimulus interval. a) Averaged psychophysiological signals. The thin black lines show the signals for each participant and odor. The colored lines represent the average responses for each specific odor, while the black line indicates the grand average across all participants. Signals were standardized at the individual participant level. b) Partial effect patterns of psychophysiological signals across 3 perceptual dimensions. The plots display the time-resolved contribution (partial effect) of each physiological signal (rows) to the 3 perceptual models (columns). Thick lines depict group-level effects (GE) with 95% pointwise confidence intervals (shaded areas), while thin lines represent individual-level effect patterns (IE) derived from factor-smooth interactions. Wald-like test statistics are provided for both levels and indicate significance across the entire smooth function (bold values indicate *P* < 0.05). To interpret these patterns, the partial effect (*y*-axis) should be viewed as the instantaneous “weight” of the physiological signal in predicting the outcome. A positive partial effect signifies that higher physiological activity (increased signal amplitude) at that specific moment drives the predicted perceptual score upward, whereas a negative effect implies that higher activity drives the score downward. This dynamic is best understood by contextualizing deviations from the average signal trend (a). For example, as the typical respiratory response involves an inhalation peak around 1.9 s post-stimulus, a positive partial effect around this time point indicates that a stronger inhalation (e.g. deeper than the norm) predicts a higher perceptual rating, while a negative effect would suggest the inverse.

Participants showed reliable psychophysiological signals after odor exposure for respiration and heart rate (Fig. 3a). Following our instruction, they inhaled with odor exposure and started exhaling after approximately 1.9 s. The respiration-corrected heart rate peaked around 1.3 s after stimulus onset, followed by a deceleration phase lasting about 3 s. Muscle activity in the corrugator reached a local maximum after 2.8 s, just before the end of the odor presentation, while zygomaticus muscle activity did not change during odor presentation and gradually increased afterwards.

We analyzed the unique group-level and individual-level effect patterns of the psychophysiological patterns on the perceptual dimensions (Fig. 3b). In the following, these effect patterns will be discussed and contextualized based on the average time course of each psychophysiological signal.

For *pleasantness*, we found statistically significant effects of group-level respiration patterns (edf [estimated degrees of freedom; summary statistic that indicates the degree of {non}linearity of the relationship. Values > 1 indicate increasing departure from a linear relationship.] = 2, *F*(2) = 12.32, *P* < 0.001). Specifically, deeper inhalation during odor presentation was associated with a more positive valence perception. In addition to this group effect, individual respiration patterns mattered for pleasantness (edf = 50.31, *F*(160) = 6.05, *P* < 0.001). Especially the habitual shaping of the initial amplitude and inhalation delay (i.e. whether a person in general tends to inhale more or less deep and more or less long) was predictive. Furthermore, tension of the corrugator muscle mattered on the group level (edf = 6.32, *F*(7.36) = 4.64, *P* < 0.001). Decreased activity around 0.8 and 3 s was associated with higher pleasantness. Individual tendency in corrugator patterns did not explain additional variance. The effect of heart rate was only significant on the individual level (edf = 13.79, *F*(160) = 0.12, *P* = 0.044). Individual participants showed heart rate patterns with a wave-like pattern around the group-level effect even though the group-level effect itself was not significant. The primary difference between these individual patterns was the timing of the most predictive moments, but no specific time point was consistently important across all participants.

For *temperature* perception, no group-level effect became significant, but individual patterns mattered for heart rate (edf = 35, *F*(160) = 0.45, *P* < 0.001), and EMG corrugator (edf = 33.89, *F*(160) = 0.33, *P* = 0.001). For heart rate, the individual heart rate patterns generally mirrored the overall group trend. This group trend, although not statistically significant, typically showed a wave-like pattern with its highest positive peak occurring around 2 s after odor onset. Individual patterns primarily differed from this group trend in the magnitude of the partial effects, with only a few participants showing patterns that were qualitatively different in shape. For the corrugator, individual patterns varied largely in terms of magnitude and response timing. These findings suggest that while participants generally exhibited consistent psychophysiological responses to warmly and coldly perceived odors, this consistency was only evident at an individual level.

*Intensity* related to respiration at the group-level (edf = 3.22, *F*(3.55) = 2.8, *P* = 0.038). Specifically, shallower inhalation and early exhalation during 1.5 to 3 s post-stimulus were associated with higher perceived odor intensity. In addition, the individual effect became statistically significant (edf = 44.51, *F*(160) = 1.9, *P* = 0.003). While participant-specific patterns varied primarily in the magnitude of peak exhalation and inhalation partial effects, response timing remained consistent. Notably, participants exhibited divergent effect directions, with, for example, shallower initial inhalation associated with higher intensity for some, but lower intensity for others. Intensity also related to corrugator (edf = 2, *F*(2) = 5.36, *P* = 0.005) and zygomaticus muscle activity (edf = 2, *F*(2) = 3.52, *P* = 0.03) on the group level and increased muscle tension on both sites during odor presentation was associated with a more intense odor perception.

## 4. Discussion

### 4.1 Primary drivers of perceptual variance

Our findings revealed variations in the explained variance across the 3 perceptual dimensions of valence, perceived temperature, and intensity, based on the presented odors, individual rating tendencies, and group level as well as individual physiological responses to the presented odor.

Across all perceptual dimensions, our models accounted for approximately 50% to 60% of the variance. While the amount of explained variance appears to be limited, the prediction of perceptual responses on the individual level is inherently bound by intraindividual variations. These variations, quantified by test–retest correlations, often do not exceed correlations of 0.7, which equates to a 49% upper bound of explainable variance (Keller et al. 2017; Trimmer et al. 2019). Given this context, our proportion of explained variances is well aligned with current research.

While the odorant itself and individual rating tendencies accounted for the most variance in all 3 perceptual dimensions, their relative contributions differed. For valence, the odorant explained approximately twice the variance of individual rating tendencies. In contrast, for temperature, both information sources contributed similarly. Notably, for intensity, this pattern reversed, with individual rating tendencies explaining nearly 5 times more variance than the odor itself. This underscores the importance of considering and evaluating interindividual differences in perceptual ratings where participants' self-selected reference points for intensity and perceived irritability likely contribute to the explained variance. In our study, we aimed for isointense odors, which may explain the limited group-level effect of odor on intensity.

### 4.2 Group-level psychophysiological signatures of odor perception

Conversely, the group-level and individual effects of respiration, heart rate, and EMG activity at the zygomaticus and corrugator sites explained considerably smaller proportions of variance compared with the odorants and rating tendencies. Nevertheless, the inclusion of physiological signals significantly improved model fit. This underscores their value in capturing subtle nuances in the response to odors that subjective ratings alone do not account for.

Group-level effects of physiological signals significantly explained variance for the prediction of the valence and intensity of an odor, suggesting a generalizable pattern of autonomous and affective physiological responses to odors. These group-level effects were primarily driven by respiration and EMG corrugator, showing significant effects for valence and intensity, aligning with previous research (Bensafi et al. 2002b; Delplanque et al. 2009; Pichon et al. 2015; Joussain et al. 2017). Specifically, increased respiration during the initial inhalation phase correlated with higher valence, while decreased respiration at the end of the stimulus presentation, corresponding to the initial exhalation, was associated with higher intensity. For EMG, the pattern was reversed, with increased initial activity being associated with higher intensity and lower valence. Interestingly, while the entire odor presentation period correlated to intensity, valence showed 2 distinct predictive time windows: one immediately after odor onset and another around odor offset. These temporally distinct responses, though similar in direction and magnitude, may reflect different underlying processes, with the initial response potentially indicating a lower-order reflexive reaction and the later response reflecting higher-order evaluative processes. This pattern mirrors findings from chemosensory event-related potentials, where early components, such as the N1 and P2, reflect the initial sensory encoding of olfactory stimuli linked to concentration and intensity (Pause et al. 1997; Pause and Krauel 2000), while a later positive component relates to the conscious appraisal of odors (Pause and Krauel 2000; Lundström et al. 2006). Contrary to our initial hypotheses, EMG zygomaticus did not correlate with valence but with intensity, where higher muscle activity during odor presentation related to higher perceived intensity. This finding aligns with a previous null finding (Delplanque et al. 2009) and may indicate a general increase in facial muscle tension in response to intense odors rather than a positive affective response. The group-level effect for heart rate did not relate to any perceptual dimension. While these results contrast with our expectation, especially for the relation to valence or pleasantness, they align with a set of studies that did not find a relation between heart rate and pleasantness (Robin et al. 1998; Djordjevic et al. 2008; He et al. 2016; Joussain et al. 2017). This may be due to the limited set of odorants presented, which do not span the full range of the pleasantness spectrum, when compared with a larger set of odors specifically selected to maximize the pleasantness range (Pichon et al. 2015).

### 4.3 Idiosyncratic and participant-specific response patterns

Beyond examining significant group-level effects of physiological signals, we explored consistent participant-specific effect patterns not captured by group-level analyses. Indeed, multiple individual effect patterns emerged, particularly for respiration (significant effects on valence and intensity), heart rate (significant effects on valence and temperature), and EMG corrugator (significant effect on temperature). Although interpreting the numerous interindividually varying response patterns is complex, examining their general trends reveals variations in magnitude (predictive strength of specific time points), delay (differences in the most predictive time points across participants), and form (divergence of individual effect patterns from the general trend). Notably, while most effect patterns exhibited all 3 properties, their dominance varied. For example, the individual effect pattern of respiration on valence primarily differed in delay, suggesting that while an increased initial inhalation was generally associated with positive valence, the precise timing of this influence varied across individuals. Conversely, the individual effect pattern of respiration on intensity showed low variation in delay and form but high variation in magnitude. Furthermore, many participants displayed opposing effect patterns for respiration in response to intensity, where some showed early deep inhalation followed by deep exhalation, while others exhibited shallower initial inhalation or even initial exhalation followed by stronger inhalation toward the end of odor presentation. These opposing patterns, while potentially predictive at the individual level, may obscure group-level effects by canceling each other out, emphasizing the importance of examining autonomous responses at both group and individual levels, or within clusters of individuals with similar response patterns.

### 4.4 Differentiating universal and idiosyncratic contributions to perception

The simultaneous examination of significant group-level and individual effect patterns offers further insights. Considering respiration and EMG corrugator, the 2 physiological signals with significant group-level effects on valence and intensity, a distinct pattern emerges. Respiration shows both a predictive general trend and incremental predictivity at the individual level. However, EMG corrugator shows no additional predictive gain at the individual level, suggesting that the model captured all relevant consistent physiological responses to odor intensity at the group level. This is notable as it indicates our modeling approach can differentiate between group-wide typical physiological responses with a potentially large universally predispositioned component (defined by effect patterns significant only at the group level), primarily idiosyncratic responses with a potentially large learning component (defined by effect patterns significant only at the individual level), and responses with shared universal and idiosyncratic influences (defined by significant effect patterns at both group and individual levels). Based on this, we infer that participants' respiratory responses to valence- and intensity-based components of odors were influenced by both a potentially reflexive typical response and an idiosyncratic response, while the EMG corrugator response was primarily reflexive and universal. This aligns with the notion that some physiological components are more universally determined (e.g. corrugator as an involuntary muscle movement), while others are more individually determined and under voluntary control (e.g. respiration showing both).

Furthermore, this approach illuminates how the same physiological signal exhibits different proportions of innate and learned components in response to varying perceptual properties of a stimulus. For instance, EMG corrugator primarily showed a group-level typical response to valence and intensity but individual-specific idiosyncratic responses to perceived temperature, indicating consistent within-participant response patterns to perceived cold or warm odors with minimal overlap across participants. This distinction between valence and intensity on the one hand, and temperature on the other, is plausible. Valence and intensity, which in this case includes perceived irritability, relate to the affective appraisal of a stimulus in terms of approach and avoidance behaviors. Consequently, a group-consistent typical response to potentially hazardous stimuli would offer a survival advantage. Conversely, temperature does not primarily drive approach or avoidance but represents a more nuanced evaluation of odor characteristics that may express itself as idiosyncratic physiological responses. This aligns with the idea that some perceptual domains are more universally determined (intensity as a basic sensation), while others, like perceived temperature as a higher-order evaluation, are more individually determined and thus more associated with experience, potentially reflecting different levels of mentalizing (high vs. low order).

### 4.5 Future directions

In this study, we utilized monomolecular odorants as stimuli, which offer a valuable approach for investigating emotional perception due to the ability of odors to modulate emotions and the high overlap of cortical structures involved in olfactory and emotional processing (Soudry et al. 2011; Kontaris et al. 2020).

However, the methodology employed here has potential applications extending beyond olfactory research to encompass broader perceptual phenomena. Examining the relationship between the same autonomous and affective physiological measures and similar perceptual properties across multiple sensory modalities, not solely olfaction, could further elucidate how specific sensory channels and their associated cognitive processes influence particular physiological responses. Additionally, signals like electrodermal activity, pupillometry, or neuroimaging methods such as functional near-infrared spectroscopy have all time-courses suitable to include in the presented methodology and may further elucidate the interplay of bodily and conscious perceptual responses. Further, 2 of our perceptual dimensions, valence and intensity, are known to be influenced by cultural differences (Ayabe-Kanamura et al. 1998; Chrea et al. 2004). Thus, it may be worthwhile to apply our methodological approach to a culturally diverse population.

### 4.6 Limitations

While our findings support the utility of assessing physiological responses to enhance the prediction of individual odor perception, several limitations warrant consideration. Although we selected a set of monomolecular odorants intended to cover a broad range of perceptual qualities, we presented only 6 distinct odorants. The limited number of odorants may restrict the generalizability of our findings to a broader range of olfactory experiences. Future research should address this by including ratings from a more extensive array of perceptually distinct odors.

While we chose a strict experimental approach, our exact setting led to some potential limitations. First, we instructed the participants to time-locked inhalation patterns. While this improves the alignment of trials within and between participants, it restricts spontaneous physiological reactions. Thus, predictive physiological response patterns found in our study may not be the same as patterns found in response to spontaneous and unprimed odor presentations. Second, while we utilized an olfactometer for millisecond-precise odor presentations, we did not assess the potential time delay introduced by the odor delivery tubes. While our methodological approach should be robust toward slight delays, the comparison between exact time points in our study with results of other studies can differ by a few milliseconds.

Additionally, while we used psychophysiological signals to predict perceptual responses in a methodological sense, we cannot infer a causal direction based on our study, i.e. if specific physiological response patterns lead to specific responses or the other way around. Even more, the specific psychophysiological response pattern may incorporate both an involuntary early physiological response that shapes the perceptual response, e.g. a reflexive early inhalation stop of highly intense or unpleasant odors, and a voluntary modulation of the physiological signal based on the percept, e.g. a long and sustained inhalation of pleasant odors. This view aligns well with the reciprocal interaction of olfactory and emotional cortical circuits that may promote bidirectional modulations (Kontaris et al. 2020).

Nevertheless, while this study provides a robust starting point and a valuable methodological framework, the transferability of our findings to more naturalistic and complex olfactory experiences, where processes such as familiarity and identification play a more significant role, is still pending and presents an important avenue for subsequent investigations. Furthermore, our results should be validated using a wider variety of distinct perceptual properties and physiological measures.

## 5. Conclusion

Our findings underscore the intricate nature of olfactory perception, which, while complex, appears to be predominantly shaped by the characteristics of the perceived odorant and individual-specific, idiosyncratic rating tendencies. The inclusion of physiological measures enriches this understanding by revealing subtle insights into latent processes involved in olfactory perception that extend beyond the influence of the odorant itself. These physiological insights can be further differentiated into universal responses, similar across participants and potentially reflecting reflexive components, and more idiosyncratic processes that are predictive for specific individuals, which may coincide with learned or experiential aspects of affective and perceptive responses. The evaluation of these universal and idiosyncratic components across various perceptual dimensions in parallel offers valuable information on their relative importance and how their influence shifts depending on the specific property being assessed, thereby providing a more nuanced and comprehensive understanding of the multifaceted nature of olfactory perception.

## Supplementary Material

bjag009_Supplementary_Data

## Data Availability

The data and source code needed to compute the nonlinear model can be found online: https://doi.org/10.5281/zenodo.17672578
