# Supplementary material for: Decoding odor responses: universal patterns and individual signatures in psychophysiology using nonlinear models
Source: Chem Senses. 2026 Mar 5;51:bjag009. doi: 10.1093/chemse/bjag009 (PMC13016868; doi:10.1093/chemse/bjag009)
Supplement: bjag009_Supplementary_Data [file bjag009_supplementary_data.docx]

Appendix

A1: Rating Distribution of the perceptual properties





***Figure A1:*** *Distribution of the perceptual ratings given on a Visual Analogue Scale, ranging from 0 (“Does not fit at all”) to 100 (“Fits very well”). Point ranges depict means and standard deviations.*

A2: Model Comparison of time-averaged and nonlinear Models

To assess if incorporating the time-course of the psychophysiological signals leads to a better model fit than standard modelling with time-averaged summary statistics, we computed three linear mixed-effects models with the three perceptual dimensions valence, temperature, and intensity as response. In alignment with the nonlinear models, we included the odorant, the test half in which the trial occurred, and a random intercept for each participant as predictors. As time-averaged counterparts for respiration and heart rate we chose the average heart rate and average amplitude across a trial as predictors. For EMG zygomaticus and EMG corrugator, we chose binary indicator variables for meaningful EMG activity as predictors. Following the toolbox Neurokit2 (Makowski et al., 2021), meaningful EMG activity was defined as EMG amplitudes exceeding one tenth standard deviation of the participant’s entire EMG signal. Model fits of the time-averaged and nonlinear models were then compared using the Akaike Information Criterion (AIC) and the Bayesian Information Criterion (BIC). For all three perceptual dimensions, the nonlinear model showed lower values, indicating a better model fit, compared to the time-averaged models (see Table A1)

***Table A1****: Model fit indices of the time-averaged and nonlinear models*

| **Response** | **AIC**  **(time-averaged)** | **AIC**  **(nonlinear)** | **BIC**  **(time-averaged)** | **BIC**  **(nonlinear)** |
| --- | --- | --- | --- | --- |
| Valence | 9062.3 | 4861.7 | 9146.1 | 5596.3 |
| Temperature | 7663.6 | 3896.1 | 7747.3 | 4853.5 |
| Intensity | 7521.9 | 3953.1 | 7605.7 | 4643.4 |

A3: Participant-wise Predictions

***Table A2:*** *Average perceptual dimension scores and model predictions for each combination of participant (ID) and odor*

| Odor | ID | Valence  Score | Valence Predicted | Temperature  Score | Temperature  Predicted | Intensity  Score | Intensity  Predicted |
| --- | --- | --- | --- | --- | --- | --- | --- |
| amycap | g07a017 | -0.869 | -0.464 | -0.482 | -0.068 | 0.881 | -0.257 |
| benzyl | g07a017 | -0.199 | -0.426 | -0.484 | -0.341 | 0.298 | -0.316 |
| carvon | g07a017 | -0.18 | 0.215 | 0.001 | -0.467 | -0.586 | -0.285 |
| decan | g07a017 | -0.022 | -0.393 | 0.06 | 0.106 | -1.369 | -0.667 |
| maltol | g07a017 | -0.176 | 0.502 | 0.624 | 0.737 | -0.806 | -0.761 |
| tolyl | g07a017 | 0.133 | -0.887 | 0.207 | -0.052 | -1.432 | -0.331 |
| amycap | g07a018 | -0.698 | -0.078 | -0.494 | -0.529 | 0.452 | 0.713 |
| benzyl | g07a018 | -0.101 | 0.177 | -0.609 | -0.833 | 0.239 | 0.588 |
| carvon | g07a018 | 2.022 | 0.833 | -1.512 | -1.055 | 1.149 | 0.828 |
| decan | g07a018 | -0.607 | -0.124 | -0.53 | -0.391 | 0.668 | 0.376 |
| maltol | g07a018 | 1.655 | 1.095 | 0.48 | 0.235 | 0.143 | 0.075 |
| tolyl | g07a018 | -1.36 | -0.511 | -0.312 | -0.495 | 0.403 | 0.583 |
| amycap | g07a019 | 0.46 | -0.059 | -0.119 | -0.102 | -0.214 | -0.115 |
| benzyl | g07a019 | -0.043 | -0.008 | -0.508 | -0.381 | 0.075 | -0.214 |
| carvon | g07a019 | 0.928 | 0.789 | -0.349 | -0.556 | 0.014 | -0.125 |
| decan | g07a019 | -0.103 | 0.034 | 0.024 | 0.027 | -0.268 | -0.526 |
| maltol | g07a019 | 0.584 | 1.01 | -0.052 | 0.608 | -0.55 | -0.605 |
| tolyl | g07a019 | -0.451 | -0.412 | 0.396 | -0.094 | -0.846 | -0.212 |
| amycap | g07a026 | 0.196 | 0.43 | 0.291 | 0.184 | 0.591 | 0.346 |
| benzyl | g07a026 | 0.997 | 0.613 | 0.261 | -0.028 | -0.096 | 0.107 |
| carvon | g07a026 | 1.506 | 1.379 | -0.513 | -0.309 | 0.166 | 0.126 |
| decan | g07a026 | 0.113 | 0.681 | 0.035 | 0.36 | -0.971 | -0.328 |
| maltol | g07a026 | 0.291 | 1.059 | 0.006 | 0.733 | 0.875 | 0.364 |
| tolyl | g07a026 | 0.684 | 0.163 | 0.827 | 0.194 | 0.412 | 0.162 |
| amycap | g07a027 | 0.227 | -0.249 | -0.155 | -0.24 | -0.405 | -0.498 |
| benzyl | g07a027 | -0.144 | -0.236 | -0.478 | -0.487 | -0.304 | -0.59 |
| carvon | g07a027 | 0.174 | 0.553 | -0.161 | -0.652 | -0.86 | -0.66 |
| decan | g07a027 | 0.016 | 0.006 | -0.344 | -0.144 | -1.856 | -1.353 |
| maltol | g07a027 | 0.317 | 0.811 | 0.614 | 0.592 | -0.821 | -1.008 |
| tolyl | g07a027 | -0.084 | -0.599 | -0.778 | -0.25 | -0.468 | -0.696 |
| amycap | g07a032 | -0.731 | 0.232 | -1.493 | -0.695 | -0.081 | -0.098 |
| carvon | g07a032 | 2.084 | 1.176 | -1.289 | -0.985 | -0.242 | -0.102 |
| tolyl | g07a032 | -0.047 | -0.043 | 0.53 | -0.406 | -0.014 | -0.113 |
| amycap | g07a244 | 0.058 | 0.17 | 0.511 | 0.051 | -0.101 | -0.071 |
| benzyl | g07a244 | 0.829 | 0.221 | -0.211 | -0.226 | -0.038 | -0.114 |
| carvon | g07a244 | 0.531 | 0.991 | -0.418 | -0.45 | -0.6 | -0.109 |
| decan | g07a244 | 0.698 | 0.276 | 0.395 | 0.214 | -0.448 | -0.487 |
| maltol | g07a244 | 0.673 | 1.278 | 0.045 | 0.761 | -0.217 | -0.626 |
| tolyl | g07a244 | -0.263 | -0.235 | 0.01 | 0.029 | -0.17 | -0.135 |
| amycap | g07a901 | 0.945 | -0.271 | 0.189 | 0.063 | 0.759 | 0.877 |
| benzyl | g07a901 | -0.857 | -0.178 | -0.466 | -0.279 | 1.196 | 0.771 |
| carvon | g07a901 | -0.785 | 0.352 | -0.085 | -0.394 | 1.515 | 0.87 |
| decan | g07a901 | -0.114 | -0.235 | 0.326 | 0.218 | 0.482 | 0.435 |
| maltol | g07a901 | 1.693 | 1.094 | 0.997 | 0.736 | -0.221 | 0.228 |
| tolyl | g07a901 | -0.625 | -0.516 | -0.706 | -0.043 | 0.142 | 0.624 |
| amycap | g07b056 | -0.165 | -0.155 | -0.223 | -0.437 | -0.183 | -0.441 |
| benzyl | g07b056 | 0.186 | -0.2 | -0.562 | -0.73 | -0.783 | -0.487 |
| carvon | g07b056 | 0.319 | 0.481 | -0.927 | -0.902 | -1.065 | -0.438 |
| decan | g07b056 | -0.474 | -0.144 | -0.206 | -0.294 | -1.041 | -0.833 |
| maltol | g07b056 | 0.19 | 0.822 | -0.149 | 0.245 | -0.412 | -0.948 |
| tolyl | g07b056 | 0.239 | -0.546 | -0.505 | -0.441 | -0.289 | -0.574 |
| amycap | g07b058 | 0.625 | -0.016 | 1.091 | 0.334 | -0.128 | 0.303 |
| benzyl | g07b058 | -0.49 | 0.051 | -0.715 | 0.027 | 0.392 | 0.223 |
| carvon | g07b058 | 0.522 | 0.653 | 0.284 | -0.13 | 0.127 | 0.295 |
| decan | g07b058 | 0.399 | 0.134 | 0.92 | 0.46 | -0.441 | -0.143 |
| maltol | g07b058 | 1.386 | 1.102 | 1.509 | 1.111 | 0.088 | -0.162 |
| tolyl | g07b058 | -0.682 | -0.435 | -0.55 | 0.313 | 0.596 | 0.232 |
| amycap | g07b062 | 0.085 | -0.415 | -0.924 | -0.757 | 0.227 | 0.541 |
| benzyl | g07b062 | -0.621 | -0.402 | -0.626 | -0.945 | 0.755 | 0.507 |
| carvon | g07b062 | 0.011 | 0.326 | -0.9 | -1.157 | 0.638 | 0.552 |
| decan | g07b062 | -0.042 | -0.31 | -0.747 | -0.569 | 0.617 | 0.075 |
| maltol | g07b062 | -0.301 | 0.523 | -0.373 | -0.025 | -0.619 | -0.046 |
| tolyl | g07b062 | 0.174 | -0.767 | -0.782 | -0.698 | 0.152 | 0.473 |
| amycap | g07b063 | -0.991 | -0.427 | -0.12 | -0.179 | -0.009 | -0.097 |
| benzyl | g07b063 | -0.797 | -0.337 | -0.1 | -0.433 | 0.024 | -0.124 |
| carvon | g07b063 | 0.45 | 0.377 | -0.959 | -0.598 | 0.304 | -0.08 |
| decan | g07b063 | -0.155 | -0.367 | -0.529 | -0.048 | -1.183 | -0.484 |
| maltol | g07b063 | 1.461 | 0.791 | 1.428 | 0.678 | -0.662 | -0.577 |
| tolyl | g07b063 | -0.631 | -0.729 | -0.54 | -0.114 | -0.007 | -0.124 |
| amycap | g07b064 | -0.479 | -0.128 | 0.125 | 0.133 | -0.235 | -0.139 |
| benzyl | g07b064 | -0.048 | 0.047 | -0.079 | -0.111 | -0.609 | -0.209 |
| carvon | g07b064 | 0.651 | 0.64 | 0.207 | -0.28 | -0.511 | -0.142 |
| decan | g07b064 | 0.565 | 0.061 | 0.062 | 0.325 | -0.368 | -0.534 |
| maltol | g07b064 | 1.311 | 1.119 | 0.974 | 0.902 | -0.259 | -0.662 |
| tolyl | g07b064 | -0.917 | -0.563 | -0.345 | 0.126 | 0.232 | -0.201 |
| amycap | g07b066 | -1.072 | -1.237 | 0.347 | 0.693 | 0.474 | 0.069 |
| benzyl | g07b066 | -1.581 | -1.062 | 0.636 | 0.383 | -0.665 | -0.111 |
| carvon | g07b066 | -1.725 | -0.499 | 0.515 | 0.278 | 0.734 | 0.115 |
| decan | g07b066 | -0.902 | -1.082 | 1.01 | 0.842 | -0.725 | -0.431 |
| maltol | g07b066 | 1.599 | 0.079 | 1.554 | 1.42 | -0.436 | -0.496 |
| tolyl | g07b066 | -1.331 | -1.337 | 0.165 | 0.6 | -0.326 | -0.146 |
| amycap | g07b067 | 1.005 | 0.528 | 0.323 | 0.133 | 0.226 | 0.673 |
| benzyl | g07b067 | -0.529 | 0.597 | 0.034 | -0.098 | 1.171 | 0.642 |
| carvon | g07b067 | 1.949 | 1.426 | -1.108 | -0.393 | 0.702 | 0.637 |
| decan | g07b067 | 1.65 | 0.644 | 0.842 | 0.324 | 0.301 | 0.278 |
| maltol | g07b067 | 1.285 | 1.753 | 0.755 | 0.849 | 0.228 | 0.163 |
| tolyl | g07b067 | -0.509 | -0.023 | 0.244 | 0.152 | 0.417 | 0.578 |
| amycap | g07b070 | -0.149 | -0.602 | -1.078 | -0.871 | 0.348 | 0.742 |
| benzyl | g07b070 | 1.387 | -0.32 | -0.655 | -1.09 | 0.265 | 0.799 |
| carvon | g07b070 | -0.546 | 0.038 | -1.19 | -1.249 | -0.059 | 0.613 |
| decan | g07b070 | -0.37 | -0.327 | -0.056 | -0.619 | 1.189 | 0.51 |
| maltol | g07b070 | -0.578 | 0.673 | -1.225 | -0.09 | 0.804 | 0.381 |
| tolyl | g07b070 | -1.243 | -1.065 | -0.195 | -0.645 | 1.383 | 0.847 |
| amycap | g07b075 | -0.179 | -0.639 | 0.759 | 0.546 | 0.092 | 0.656 |
| benzyl | g07b075 | 0.068 | -0.599 | -0.63 | 0.258 | 0.879 | 0.753 |
| carvon | g07b075 | 0.643 | 0.149 | -0.852 | 0.078 | 0.346 | 0.639 |
| decan | g07b075 | -1.4 | -0.718 | 1.142 | 0.775 | 1.052 | 0.53 |
| maltol | g07b075 | -0.19 | 0.432 | 1.59 | 1.303 | 0.793 | 0.432 |
| tolyl | g07b075 | -1.219 | -1.084 | 1.352 | 0.649 | 0.401 | 0.527 |
| amycap | g07b080 | 0.063 | -0.122 | -0.058 | 0.048 | 0.777 | 0.702 |
| benzyl | g07b080 | -0.291 | -0.065 | 0.511 | -0.236 | 0.172 | 0.637 |
| carvon | g07b080 | 0.536 | 0.626 | 0.591 | -0.342 | 0.932 | 0.687 |
| decan | g07b080 | -0.558 | -0.087 | -0.225 | 0.17 | 0.638 | 0.325 |
| maltol | g07b080 | 1.725 | 1.102 | -0.113 | 0.706 | -0.287 | 0.153 |
| tolyl | g07b080 | -0.305 | -0.496 | -0.076 | 0.056 | 0.549 | 0.652 |
| amycap | g07b982 | -0.231 | -0.112 | -0.03 | 0.007 | 0.979 | 0.983 |
| benzyl | g07b982 | -1.205 | -0.361 | -0.035 | -0.198 | 1.271 | 0.968 |
| carvon | g07b982 | 1.976 | 0.892 | 0.154 | -0.398 | 0.566 | 0.825 |
| decan | g07b982 | -0.102 | -0.107 | -0.22 | 0.102 | 0.805 | 0.597 |
| maltol | g07b982 | 0.787 | 1.139 | 0.37 | 0.755 | 0.416 | 0.387 |
| tolyl | g07b982 | -0.051 | -0.223 | 0.114 | 0.006 | 0.521 | 0.748 |
| amycap | g07b984 | 0.649 | 0.081 | 0.605 | 0.395 | -0.057 | 0.032 |
| benzyl | g07b984 | -0.073 | 0.062 | -0.218 | 0.067 | -0.305 | 0.003 |
| carvon | g07b984 | 0.579 | 0.766 | 0.116 | -0.063 | -0.405 | -0.003 |
| decan | g07b984 | 0.089 | 0.112 | -0.223 | 0.554 | -0.253 | -0.367 |
| maltol | g07b984 | 1.857 | 1.129 | 1.091 | 1.157 | 0.179 | -0.454 |
| tolyl | g07b984 | -0.986 | -0.382 | 0.753 | 0.414 | 0.303 | -0.023 |
| amycap | g07c082 | -0.605 | -0.371 | 0.061 | 0.148 | 0.411 | -0 |
| benzyl | g07c082 | -0.325 | -0.237 | -0.103 | -0.191 | 0.138 | -0.124 |
| carvon | g07c082 | 0.957 | 0.504 | -0.515 | -0.349 | -0.388 | -0.111 |
| decan | g07c082 | -0.266 | -0.216 | 0.27 | 0.245 | -0.649 | -0.535 |
| maltol | g07c082 | 1.064 | 0.997 | 0.976 | 0.81 | -1.002 | -0.704 |
| tolyl | g07c082 | -0.805 | -0.642 | 0.06 | 0.089 | -0.182 | -0.219 |
| amycap | g07c083 | -0.784 | -0.633 | 0.589 | 0.335 | 0.413 | 0.615 |
| benzyl | g07c083 | -0.769 | -0.531 | 0.089 | 0.078 | 0.316 | 0.449 |
| carvon | g07c083 | 0.331 | 0.195 | -0.769 | -0.094 | 0.07 | 0.45 |
| decan | g07c083 | -0.653 | -0.582 | 0.225 | 0.507 | 0.903 | 0.245 |
| maltol | g07c083 | 0.692 | 0.593 | 1.215 | 1.126 | 0.29 | 0.012 |
| tolyl | g07c083 | -0.651 | -0.91 | 1.011 | 0.39 | 0.176 | 0.399 |
| amycap | g07c096 | 0.305 | 0.207 | 0.454 | 0.287 | -0.692 | -0.63 |
| benzyl | g07c096 | 2.076 | 0.263 | -1.149 | -0.001 | -0.146 | -0.721 |
| carvon | g07c096 | 1.02 | 0.679 | -1.623 | -0.378 | 0.689 | -0.502 |
| decan | g07c096 | -0.143 | 0.239 | 1.474 | 0.595 | -1.476 | -1.077 |
| maltol | g07c096 | -0.068 | 0.977 | 1.25 | 0.935 | -1.558 | -1.059 |
| tolyl | g07c096 | -0.789 | -0.235 | 1.415 | 0.565 | -1.408 | -0.773 |
| amycap | g07c098 | -0.199 | 0.018 | -0.227 | -0.591 | 0.135 | 0.457 |
| benzyl | g07c098 | -0.419 | 0.071 | -1.281 | -0.876 | 0.221 | 0.36 |
| carvon | g07c098 | 1.999 | 0.907 | -1.13 | -1.058 | 0.55 | 0.422 |
| decan | g07c098 | 0.277 | 0.085 | -0.176 | -0.389 | 0.096 | 0.076 |
| maltol | g07c098 | 0.188 | 1.127 | 0.186 | 0.179 | -0.002 | -0.049 |
| tolyl | g07c098 | -0.614 | -0.364 | -0.862 | -0.593 | 0.679 | 0.391 |
| amycap | g07c105 | -0.091 | -0.296 | 0.503 | 0.249 | -0.056 | -0.215 |
| benzyl | g07c105 | -0.893 | -0.312 | 0.283 | 0.027 | 0.171 | -0.215 |
| carvon | g07c105 | 0.736 | 0.599 | -0.247 | -0.267 | -1.015 | -0.341 |
| decan | g07c105 | 0.54 | -0.006 | 0.367 | 0.367 | -0.825 | -0.683 |
| maltol | g07c105 | 1.186 | 0.948 | 0.208 | 0.951 | -0.628 | -0.8 |
| tolyl | g07c105 | -1.074 | -0.597 | 0.209 | 0.231 | -0.174 | -0.366 |
| amycap | g07c106 | -0.742 | -0.579 | -0.054 | -0.125 | 1.142 | 0.445 |
| benzyl | g07c106 | -1.246 | -0.491 | -0.458 | -0.442 | -0.458 | 0.163 |
| carvon | g07c106 | -0.195 | 0.115 | -1.077 | -0.54 | 0.241 | 0.447 |
| decan | g07c106 | 0.058 | -0.448 | -0.135 | -0.011 | 0.279 | -0.127 |
| maltol | g07c106 | 1.551 | 0.652 | 1.276 | 0.637 | -0.653 | -0.136 |
| tolyl | g07c106 | -1.024 | -1.054 | -0.057 | -0.035 | 0.635 | 0.522 |
| amycap | g07c109 | -0.976 | -0.461 | -1.017 | -0.626 | 0.603 | 0.618 |
| benzyl | g07c109 | -0.991 | -0.502 | -0.804 | -0.854 | 0.718 | 0.608 |
| carvon | g07c109 | 1.55 | 0.342 | -0.866 | -1.027 | -0.056 | 0.53 |
| decan | g07c109 | -0.938 | -0.484 | -0.802 | -0.386 | 0.459 | 0.253 |
| maltol | g07c109 | 1.425 | 0.682 | 0.981 | 0.207 | 0.379 | 0.15 |
| tolyl | g07c109 | -1.315 | -0.901 | -0.827 | -0.579 | 0.545 | 0.533 |
| amycap | g07c114 | -0.676 | -0.409 | -0.118 | -0.162 | 0.285 | 0.07 |
| benzyl | g07c114 | -0.588 | -0.344 | 0.047 | -0.332 | -0.583 | -0.003 |
| carvon | g07c114 | -0.634 | 0.415 | -0.948 | -0.553 | 0.019 | 0.033 |
| decan | g07c114 | 0.848 | -0.274 | -0.169 | 0.041 | -0.421 | -0.355 |
| maltol | g07c114 | 1.354 | 0.813 | 1.097 | 0.766 | -0.495 | -0.458 |
| tolyl | g07c114 | -0.676 | -0.711 | -0.159 | -0.104 | 0.294 | -0.034 |
| amycap | g07c116 | 0.719 | 0.386 | -0.281 | -0.278 | 0.257 | 0.019 |
| benzyl | g07c116 | 0.066 | 0.281 | -0.546 | -0.548 | -0.769 | -0.068 |
| carvon | g07c116 | 0.879 | 1.112 | -0.368 | -0.728 | 0.666 | -0.005 |
| decan | g07c116 | 0.237 | 0.364 | 0.253 | -0.051 | -0.702 | -0.435 |
| maltol | g07c116 | 1.285 | 1.424 | 0.031 | 0.46 | 0.006 | -0.52 |
| tolyl | g07c116 | 0.545 | 0.005 | -0.525 | -0.286 | -1.115 | -0.076 |
| amycap | g07c117 | 0.01 | -0.404 | -0.375 | -0.23 | 0.695 | 0.203 |
| benzyl | g07c117 | -0.312 | -0.335 | -0.931 | -0.661 | 0.363 | 0.08 |
| carvon | g07c117 | -0.2 | 0.349 | -0.255 | -0.627 | -0.038 | 0.213 |
| decan | g07c117 | -0.74 | -0.325 | -0.204 | -0.13 | -0.74 | -0.3 |
| maltol | g07c117 | 0.96 | 0.765 | 0.809 | 0.512 | -0.519 | -0.365 |
| tolyl | g07c117 | -0.544 | -0.835 | -0.558 | -0.201 | 0.237 | 0.199 |
| amycap | g07c120 | -0.451 | -0.49 | 0.639 | 0.699 | 0.07 | 0.203 |
| benzyl | g07c120 | -0.351 | -0.444 | 0.6 | 0.432 | 0.268 | 0.127 |
| carvon | g07c120 | 0.096 | 0.268 | 0.766 | 0.245 | 0.105 | 0.185 |
| decan | g07c120 | -0.655 | -0.491 | 0.668 | 0.837 | 0.016 | -0.195 |
| maltol | g07c120 | 0.681 | 0.63 | 1.084 | 1.393 | -0.439 | -0.31 |
| tolyl | g07c120 | -0.709 | -0.838 | 0.551 | 0.698 | 0.056 | 0.111 |
| amycap | g07c925 | -1.23 | -0.707 | -0.565 | -0.099 | 0.565 | 0.572 |
| benzyl | g07c925 | -0.315 | -0.651 | -0.595 | -0.228 | 0.859 | 0.497 |
| carvon | g07c925 | 0.236 | 0.089 | -0.259 | -0.43 | 0.231 | 0.508 |
| decan | g07c925 | -1.477 | -0.721 | 0.29 | 0.165 | 0.57 | 0.174 |
| maltol | g07c925 | 0.86 | 0.425 | 0.907 | 0.792 | -0.65 | 0.04 |
| tolyl | g07c925 | -0.797 | -1.088 | 0.408 | 0.072 | 0.759 | 0.504 |
| amycap | g07d137 | -1.136 | -0.882 | 0.081 | 0.102 | 0.158 | 0.215 |
| benzyl | g07d137 | -1.317 | -0.9 | 0.544 | -0.126 | 0.124 | 0.143 |
| carvon | g07d137 | 0.379 | -0.07 | -1.222 | -0.368 | 0.48 | 0.197 |
| decan | g07d137 | -0.999 | -0.893 | 0.374 | 0.333 | -0.511 | -0.226 |
| maltol | g07d137 | 0.76 | 0.21 | 0.286 | 0.866 | -0.634 | -0.368 |
| tolyl | g07d137 | -1.478 | -1.347 | 0.804 | 0.235 | 0.552 | 0.184 |
| amycap | g07d140 | -0.542 | -0.423 | 0.358 | 0.234 | 0.47 | 0.358 |
| benzyl | g07d140 | 0.154 | -0.324 | 0.682 | -0.002 | -0.097 | 0.287 |
| carvon | g07d140 | 0.852 | 0.433 | -1.201 | -0.268 | 0.877 | 0.395 |
| decan | g07d140 | -0.182 | -0.293 | -0.166 | 0.394 | -0.304 | -0.002 |
| maltol | g07d140 | 0.011 | 0.707 | 0.768 | 0.97 | -0.045 | -0.14 |
| tolyl | g07d140 | -0.688 | -0.753 | 0.576 | 0.237 | 0.311 | 0.273 |
| amycap | g07d141 | -0.785 | -0.25 | -0.69 | -0.155 | 0.821 | 0.412 |
| benzyl | g07d141 | 0.11 | -0.125 | -0.412 | -0.329 | 0.448 | 0.322 |
| carvon | g07d141 | 1.814 | 0.666 | -0.635 | -0.605 | -0.166 | 0.438 |
| decan | g07d141 | -0.649 | -0.196 | 1.021 | 0.153 | -1.535 | -0.053 |
| maltol | g07d141 | 1.28 | 0.995 | 1.486 | 0.675 | -0.113 | -0.103 |
| tolyl | g07d141 | -0.777 | -0.511 | -0.744 | -0.151 | 1.163 | 0.423 |
| amycap | g07d145 | 0.64 | -0.017 | 0.355 | -0.022 | -1.689 | -0.441 |
| benzyl | g07d145 | -0.028 | -0.059 | -0.551 | -0.333 | -0.527 | -0.268 |
| carvon | g07d145 | -0.104 | 0.607 | -0.518 | -0.503 | 0.265 | -0.203 |
| decan | g07d145 | 0.351 | 0.025 | 0.092 | 0.167 | 0.653 | -0.726 |
| maltol | g07d145 | 0.696 | 0.994 | 1.141 | 0.73 | -1.09 | -0.74 |
| tolyl | g07d145 | 0.167 | -0.465 | -0.544 | -0.068 | 0.119 | -0.302 |
| amycap | g07d150 | -0.859 | -0.27 | 0.282 | 0.107 | 0.2 | 0.082 |
| benzyl | g07d150 | -0.26 | -0.175 | 0.004 | -0.177 | -0.049 | 0.027 |
| carvon | g07d150 | 1.53 | 0.818 | -0.606 | -0.353 | -0.413 | 0.008 |
| decan | g07d150 | -0.393 | -0.11 | 0.001 | 0.338 | -0.345 | -0.282 |
| maltol | g07d150 | 1.477 | 1.328 | 1.007 | 0.972 | -0.026 | -0.402 |
| tolyl | g07d150 | -0.553 | -0.591 | 0.29 | 0.151 | 0.13 | 0.009 |
| amycap | g07d156 | -0.614 | -0.466 | -0.549 | -0.281 | 0.068 | 0.181 |
| benzyl | g07d156 | -0.652 | -0.442 | -1 | -0.547 | 0.658 | 0.048 |
| carvon | g07d156 | -0.06 | 0.227 | -0.966 | -0.757 | 0.657 | 0.06 |
| decan | g07d156 | -0.241 | -0.418 | 0.598 | -0.074 | -0.253 | -0.248 |
| maltol | g07d156 | 0.977 | 0.622 | 1.287 | 0.52 | -0.91 | -0.427 |
| tolyl | g07d156 | -0.806 | -0.919 | -0.795 | -0.337 | -0.578 | 0.055 |
| amycap | g07d158 | -0.178 | -0.292 | -0.584 | -0.227 | -0.272 | -0.155 |
| benzyl | g07d158 | 0.269 | -0.307 | -0.996 | -0.509 | -1.042 | -0.223 |
| carvon | g07d158 | 0.195 | 0.417 | 0.574 | -0.493 | 0.187 | -0.113 |
| decan | g07d158 | -0.762 | -0.298 | -0.174 | -0.003 | -0.576 | -0.592 |
| maltol | g07d158 | 0.583 | 0.808 | 0.474 | 0.604 | -0.727 | -0.721 |
| tolyl | g07d158 | -0.712 | -0.826 | -0.237 | -0.208 | 0.629 | -0.181 |
| amycap | g07d924 | -0.196 | -0.158 | -0.309 | -0.253 | 0.766 | 0.975 |
| benzyl | g07d924 | 0.047 | 0.003 | -0.447 | -0.483 | 1.012 | 0.882 |
| carvon | g07d924 | 0.997 | 0.885 | -0.345 | -0.655 | 0.563 | 0.896 |
| decan | g07d924 | 0.164 | -0.068 | -0.181 | -0.074 | 0.985 | 0.56 |
| maltol | g07d924 | 1.655 | 1.055 | 0.388 | 0.499 | 0.293 | 0.443 |
| tolyl | g07d924 | -1.409 | -0.49 | -0.294 | -0.23 | 1.182 | 0.859 |
| amycap | g07d925 | 0.25 | 0.287 | 1.026 | 0.629 | -0.238 | -0.239 |
| benzyl | g07d925 | 1.444 | 0.393 | 0.719 | 0.26 | -0.36 | -0.377 |
| carvon | g07d925 | -0.896 | 0.76 | 0.032 | 0.065 | 0.387 | -0.218 |
| decan | g07d925 | 0.256 | 0.24 | 0.535 | 0.649 | -1.132 | -0.704 |
| maltol | g07d925 | 1.841 | 1.539 | 1.642 | 1.379 | -0.664 | -0.698 |
| tolyl | g07d925 | -0.347 | -0.082 | -0.297 | 0.509 | -0.43 | -0.362 |
